# The introduction of electronic prescribing in the Orchard clinic-a QI project

**DOI:** 10.1192/bjo.2021.581

**Published:** 2021-06-18

**Authors:** Hannah Sayeed, Fionnbar Lenihan

**Affiliations:** 1 ST6 Forensic Psychiatry, the Orchard clinic; 2 St Bricin's Military Hospital

## Abstract

**Aims:**

Hospital Electronic Prescribing and Medicines Administration (HEPMA) system successfully rolled out in July, 2020 at the Orchard clinic as the first site in NHS Lothian. The initial aim was to collect some “pre-HEPMA” and “post-HEPMA” data to look at “staff's attitudes to a new IT system, and does that change with successful implementation of it?” in the form of a survey. In the light of the findings of the pre-HEPMA staff survey, it became the QI project (as above).

This aimed to look at both qualitative data; in the form of a staff attitudes survey towards a new IT system and quantitative data; to measure benefits of its implementation and to address issues raised by staff in the survey in the form of an audit in both pre and post HEPMA cycles.

**Method:**

Two cycles were completed as follows:

1. Pre-HEPMA cycle in March, 2020:

Survey: Questionnaires asking question re- own IT skills, preparation and expectations of outcome of its implementation.

Audit: Measured time taken to write and re-write paper prescriptions. Proforma filled by staff to measure time taken to log on and other IT related issues.

2. Post-HEPMA cycle in October, 2020:

Survey: Replicated above questions re-own IT skills, support during and after launch, disaster recovery and views about actual outcome of its implementation.

Audit: Replicated to measure time taken to complete electronic prescriptions.

**Result:**

24 staff questionnaires returned in both cycles. Staff felt more confident in their own IT skills, training and hence competence to use HEPMA; more supported, more confident about contingency plans and HEPMA to be more beneficial than initially anticipated. Overall, actual perceived success of (91%) compared to anticipated success (71%).

The majority issues raised via the first survey were felt to be addressed. Time to log was on average

The audit showed a clear benefit in terms of clinical time saving, e.g. daily clinical time spent writing prescription reduced from an average 45 to 6 minutes with HEPMA.

The quality of documentation on the prescriptions remained unchanged.

**Conclusion:**

Staff's attitude towards IT does change after successful implementation of a new IT system. But IT issues make the whole process laborious.

There was evident benefits realization with an electronic prescribing system compared to paper prescriptions.

I was awarded “Employee of the month” for this project which I also presented at the NHS Lothian grand round (>150 attendees) for dissemination and future replication.

